# Feasibility of a Wearable Reflectometric System for Sensing Skin Hydration

**DOI:** 10.3390/s20102833

**Published:** 2020-05-16

**Authors:** Raissa Schiavoni, Giuseppina Monti, Emanuele Piuzzi, Luciano Tarricone, Annarita Tedesco, Egidio De Benedetto, Andrea Cataldo

**Affiliations:** 1 Department of Engineering for Innovation, University of Salento, 73100 Lecce, Italy; raissa.schiavoni@studenti.unisalento.it (R.S.); giuseppina.monti@unisalento.it (G.M.); luciano.tarricone@unisalento.it (L.T.); 2 Department of Information Engineering, Electronics and Telecommunications (DIET), Sapienza University of Rome, 00184 Rome, Italy; emanuele.piuzzi@uniroma1.it; 3 Université de Bordeaux, 33076 Bordeaux, France; annarita.tedesco@u-bordeaux.fr; 4 Department of Information Technology and Electrical Engineering (DIETI), University of Naples Federico II, 80125 Naples, Italy; egidio.debenedetto@unina.it

**Keywords:** medical services, microwave measurements, biomedical sensor, health 4.0, dielectric permittivity, patient monitoring, remote monitoring, skin hydration, microwave reflectometry, time domain reflectometry

## Abstract

One of the major goals of Health 4.0 is to offer personalized care to patients, also through real-time, remote monitoring of their biomedical parameters. In this regard, wearable monitoring systems are crucial to deliver continuous appropriate care. For some biomedical parameters, there are a number of well established systems that offer adequate solutions for real-time, continuous patient monitoring. On the other hand, monitoring skin hydration still remains a challenging task. The continuous monitoring of this physiological parameter is extremely important in several contexts, for example for athletes, sick people, workers in hostile environments or for the elderly. State-of-the-art systems, however, exhibit some limitations, especially related with the possibility of continuous, real-time monitoring. Starting from these considerations, in this work, the feasibility of an innovative time-domain reflectometry (TDR)-based wearable, skin hydration sensing system for real-time, continuous monitoring of skin hydration level was investigated. The applicability of the proposed system was demonstrated, first, through experimental tests on reference substances, then, directly on human skin. The obtained results demonstrate the TDR technique and the proposed system holds unexplored potential for the aforementioned purposes.

## 1. Introduction

Health 4.0 is a concept derived from the Industry 4.0 paradigm and, as such, it shares the same design principles, such as interoperability, virtualization, decentralization, real-time capability, service orientation, and modularity [1]. One of the major impacts expected from Health 4.0 is the personalization of health and care, next to real time, for patients [2]. In this regard, wearable sensing devices are key factors for revolutionizing healthcare diagnostics and delivery [3,4]. Wearable devices may enable personalized preventative care, by exploiting the early detection of vital sign anomalies in patient to improve healthcare outcomes [5,6] and to allow continuous and remote monitoring of these values [7]. There are numerous biomedical parameters that can be monitored through wearable, portable sensing solutions: electrocardiogram, oxygen saturation, heart rate, blood pressure. For these biomedical parameters, there are a number of low-cost, wearable devices that are commercially available.

On the other hand, wearable technological solutions for monitoring skin hydration are not as consolidated. In fact, as detailed in the following section, in the literature, different sensors and techniques have been developed for monitoring skin hydration. However, most of the state-of-the-art skin hydration measuring devices are not wearable; hence, they are unsuitable for real-time use and the patient has to stop their activities to carry out the measurement. These devices cannot be used, for example, in monitoring sports activities, or by patients with walking difficulties who prefer to have a wearable device, or by people who are unable to use electronic equipment to carry out autonomously the measurements.

In this context, microwave-based measurement systems represent a convenient solution for noninvasive monitoring of hydration, mostly thanks to the possibility of adapting the measurement approach to the specific application context. In microwave techniques, skin hydration measurements rely on the strong relationship between dielectric properties and water content of biological tissues [8]. Starting from these considerations, this work addresses the feasibility of an innovative wearable device that monitors skin hydration through a time-domain reflectometry (TDR)-based system. In particular, the proposed system relies on the combined use of TDR and of a wearable SE element (fabricated on a printed circuit board) to assess in real-time skin hydration. This monitoring system relies on measuring dielectric permittivity and electrical conductivity variations of the skin to infer the hydration status. In fact, the composition and metabolic activity of the skin alter its electrical properties: increasing (or decreasing) the water content of the skin increases (or decreases) the conductivity and the dielectric permittivity of the skin.

In addition to real-time measurements, TDR offers several other advantages, such as low cost, portability, possibility of remote control and high customization. Furthermore, through multiplexing, it is possible to connect one single TDR measuring device to a number of probes; in this way, it would be possible to monitor simultaneously different body parts. In the following sections, after describing the TDR-based wearable monitoring system, the results of the experimental characterization are presented and discussed in detail.

The present work is organized as follows. Section 2 addresses, in more detail, the rationale behind this work and provides an overview of state-of-the-art solutions for monitoring skin hydration. Section 3 describes the basic theoretical background behind the proposed system and the used experimental setup. Section 4 introduces to the experiments whose results are reported in Section 5. Finally, conclusions and future work are outlined in Section 6.

## 2. Rationale of the Work and Review of State-of-the-Art Methods for Monitoring Human Hydration

Skin hydration plays an important role in the analysis of various diseases and determines the effectiveness of medical therapies. In fact, there are several situations in which it is necessary to monitor skin hydration: dermatological diseases represent the major field of application of a system of this type. A number of skin conditions last a long time and some may start in childhood and continue into adulthood. Atopic dermatitis is a common, complex disease that can affect the quality of life of patients and families to a significant degree. In addition, this problem primarily affects children with an important increase of Transepidermal water loss (TEWL) leading to significant dehydration status of skin [9,10]. For this, a wearable monitoring skin hydration system represents the fundamental device to help mothers in the constant control on the kid’s sensitive skin, in order to supervise positive effects of the medical treatment and collaborate optimally with doctors. In addition to this one, dermatological illnesses that share the presence of skin dehydration also include ichthyosis [11], psoriasis [12] and eczema [13].

A skin hydration sensor can be very useful in many other contexts: consider people employed in working environments characterized by the continuous presence of cold, wind, UV rays, low humidity or pollution. These factors easily alter the hydration state of the skin [14,15,16] causing severe damage in its natural barrier function. There are many working environments characterized by at least one condition of this type; consequently, monitoring the hydration of the skin could certainly be useful to intervene promptly before the skin damage becomes more serious and requires more important pharmacological treatments. A different scenario, but equally important, regards menopausal women affected by a decrease in hormone estrogen that causes various skin alterations as dehydration of stratum corneum [17,18]. Certainly, also stress, smoke and alcohol can cause deteriorating effects on the skin and its hydration status [19]. Attention is required also for many elderly people that have fragile, thin and dry skin due to cellular aging. Hydration skin measurement is also useful in cosmetology, to monitor the health and hydration of the skin subjected to chemical, biochemical, physical or surgical treatments which can stress it before obtaining the desired effects [20,21]. All these cases demonstrate very clearly the primary importance of a skin hydration sensing system to detect problems in skin hydration that could not be diagnosed through techniques that measure total body water (as bioimpedance): a check of total body water, in fact, will give no useful information because a subject may be normally hydrated inside but he could suffer from dermatological diseases or he might have to do with some of the aforementioned conditions, which affect his state of superficial skin hydration. In addition, another strong point of this system is the possibility of carrying out measurements on localized parts of the body (arms, legs, etc.) where necessary.

Finally, studies have demonstrated the correlation between skin hydration and total body water level. In fact, diseases such as hypothyroidism, scleroderma and diabetes cause a significant decrease in body water level, which generally reflects into dry skin: many studies, in fact, show a close correlation between skin hydration and total body water level [22,23,24]. This suggests that while skin hydration is contingent to several factors (nonetheless to external/environmental ones), it is also an indicator of possible illness that brings a global dehydration which can manifest also in dry skin.

In such a context, the major purpose of the proposed sensing system is to monitor skin hydration in those situations that directly affect the skin, as explained above, but also in the cases of more complex diseases affecting the whole organism, our system may play an important role. In this context, in fact, a skin hydration sensor can be useful as indicator that pharmacological therapy is not functioning properly, or as aid to suffering people to monitor their symptoms. For all these reasons, it is important to develop a measurement system for sensing skin hydration status and, also in cases of at-home patients, for allowing the doctor to check this parameter remotely.

The sensing technology of state-of-the-art systems, typically, relies on confocal Raman spectroscopy [25] which detects skin hydration through the analysis of the light that is scattered by the tissue. However, this method is quite expensive. Methods such as near-IR spectroscopy [26], or terahertz spectroscopy [27] have the same cost problem. Skin hydration levels can also be characterized through measurements of skin thermal conductivity [28]; nevertheless, this approach is effective only in predicting the depth of a burn through the analysis of the tissue water content and thermal properties. Recently, a system for hydration level estimation in the human body using the galvanic skin response has been presented in [29]. However, most devices are based on skin impedance measurements [30,31] through the placement of two electrodes on the skin: an increase in hydration corresponds to a decrease in skin impedance due to an increase in conductivity and dielectric constant. Other methods are based on bio-impedance measurements [32]: two or more electrodes placed on the skin are used to inject a controlled current and to measure the resulting voltage. Nevertheless, despite the large use of these procedures, the related measurement accuracy is significantly influenced by various effects such as by the variations of some physiological and environmental parameters. Other monitoring systems use a capacitive principle [33,34]: the stratum corneum is essentially a dielectric medium and its capacitance may change with the distribution of water in the skin, which directly affects the electrical field penetration into skin. In all of these types of systems, the measurement depth depends on the geometrical factors of the measurement probe or sensing element (SE). This means that measured hydration may change between the devices since there is a concentration gradient of water in the stratum corneum. Recent developments have also led to the creation of tattoos for monitoring skin hydration [35]; however, these types of systems have a short lifetime and they are not suitable for long-term skin hydration monitoring. Clearly, this review does not take into account methods that perform in vivo measurements by directly extracting fluids from the skin through microneedles. In fact, non-invasiveness is of the utmost importance for remote patient monitoring.

## 3. Materials and Methods

### 3.1. Theoretical Background

TDR is an electromagnetic (EM) measurement technique typically used for monitoring purposes [36,37,38,39]. In TDR measurements, the EM stimulus is usually a step-like voltage signal that propagates along the SE, inserted or in contact with the system to be monitored. The signal travels along the SE and any impedance variation causes the partial reflection of the propagating signal. The ratio between the amplitude of the reflected signal, $v_{ref}\left(t\right)$, and the amplitude of the generated signal, $v_{inc}\left(t\right)$, gives the value of the reflection coefficient in time domain, ρ(t).

(1) $$\rho \left(t\right)=\frac{v_{ref}\left(t\right)}{v_{inc}\left(t\right)}$$

where −1≤ρ(t)≤1. Typically, the direct output of a TDR measurement is a reflectogram, which shows ρ as a function of apparent distance, $d_{app}$. This quantity is related to the dielectric characteristics of the material in which the TDR signal is propagating, according to the following equation:

(2) $$d_{app}≅d\times \sqrt{\varepsilon_{r}}$$

where *d* is the physical distance travelled by the propagating TDR signal; and $\varepsilon_{r}$ is the relative dielectric permivitty of the material in which the SE in inserted.

**Figure 1:**
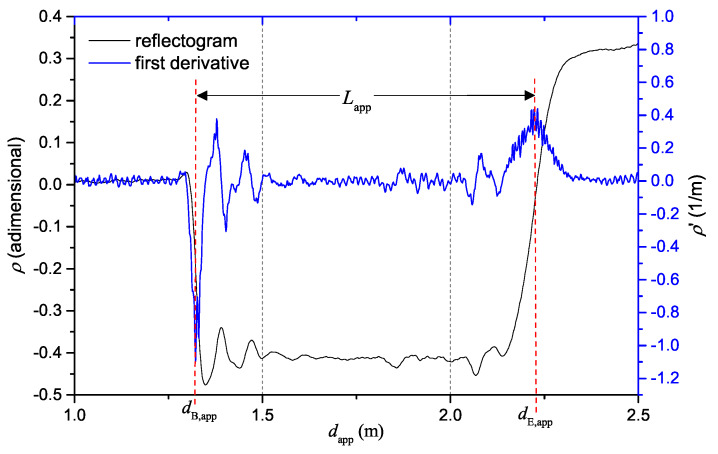
Example of a TDR reflectogram and of its first derivative for the evaluation of the apparent length of the SE.

For the sake of example, let us consider Figure 1, which shows a typical reflectogram of a probe with physical length *L*, inserted in a material with relative dielectric permittivity, $\varepsilon_{r}$.

In $d_{B,app}$, there is a steep variation of ρ(t): this variation corresponds to the beginning section of the SE. Then, the TDR signal travels along the SE, until it reaches the end of the SE, at $d_{E,app}$. At this point, the signal senses another significant variation of electrical impedance, and is reflected back towards the TDR instrument. It can be seen that the end of the SE provokes another steep variation of ρ(t). Hence, the so-called apperent length of the SE, $L_{app}$, is evaluated as

(3) $$L_{app}=d_{E,app}-d_{B,app}$$

To improve accuracy, $L_{app}$ can be evaluated from the derivative of the TDR reflectogram, which typically exhibits prominent peaks in correspondence of the probe-beginning and probe-end sections [40]. The blue curve of Figure 1 shows the first derivative of the reflectogram.

In practical applications, by evaluating the apparent length of the SE inserted in (or in contact with) a material under test, it is possible to retrieve the value of $\varepsilon_{r}$ from Equation (2).

From the analysis of the TDR reflectogram at “long distance” (i.e., for long values of $d_{app}$), it is also possible to retrieve useful qualitative information associated to the static electrical conductivity, $\sigma_{0}$ [41]. Generally, analyzing the long-distance portion of a TDR reflectogram, ρ reaches a steady state. In such condition, the lower the steady-state value of ρ, the higher the value of the static conductivity, $\sigma_{0}$.

### 3.2. Experimental Setup

**Figure 2:**
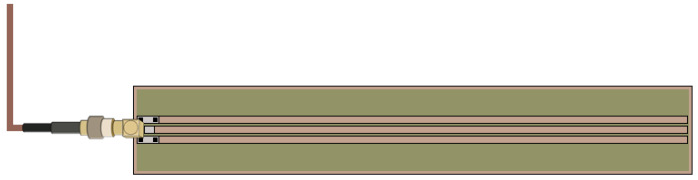
Schematization of the configuration of the used trifilar planar SE.

For the TDR measurements, a 13 cm-long trifilar ground-source-ground (GSG) planar SE was fabricated on a printed circuit board (PCB). The connection to the TDR instrument was made through a SMA connector, as shown in Figure 2. The fingers of the SE are made of copper, and they were obtained by milling the metallized PCB layer. They have a width of 2 mm with a spacing of 1 mm. This structure guarantees a good adherence to the body (and/or to the material under test). Additionally, although the prototype presented in this paper is implemented through a rigid standard FR4-PCB substrate, the proposed sensor can be easily reproduced on flexible supports (for example, by using conductive inks) and therefore it is suitable to be integrated into wearable accessories.

TDR measurements were carried out through the Campbell-Scientific TDR200, a low-cost portable measuring instrument, with approximate dimensions 22 cm ×5 cm × 11 cm. The rise time of the generated signal is approximately 200 ps, which corresponds to a frequency bandwidth of around 1.7 GHz.

Experiments were carried out by connecting the SE to the TDR200 output port through a coaxial cable. In turn, the TDR200 was connected to a laptop (through a USB cable) for data acquisition and processing.

### 3.3. Frequency-Domain Analysis

It is worth noting that for this specific application, a useful strategy is also to resort to a combined time domain/frequency domain (TD/FD) approach [42]. In this case, measurements are carried out in TD and the corresponding FD-information is retrieved through appropriate processing of the acquired data, thus “uncovering” additional information. TDR instrumentation is usually less expensive than instruments operating in frequency domain and for this reason, it represents a powerful tool for guaranteeing at the same time low cost of the setup and adequate measurement accuracy. For this purpose, as detailed in the following section, the TD results were transformed into FD to retrieve the corresponding frequency-dependent reflection scattering parameters, $S_{11}\left(f\right)$, through the following equation:

(4) $$S_{11}\left(f\right)=\frac{DFT[\rho (t)]}{DFT[\rho_{i}\left(t\right)]}$$

where $\rho_{i}\left(t\right)$ is the TDR reflectogram when the sensing element is not connected to the TDR measuring instrument, and DFT indicates the discrete Fourier transform.

To obtain accurate FD data, also a SOL calibration (short, open and load) was performed; then, the scattering parameter ($S_{11}\left(f\right)$) was extrapolated starting from the TDR reflectograms as detailed in [40,42].

## 4. Description of the Experiments

For validating the proposed system, TDR measurements were carried out on with the SE in the following conditions:

1. immersed in ultrapure water and sugar solutions;
2. immersed in saline solutions with sugar;
3. placed on human foreskin arm; and
4. placed in contact with a sample of animal hide.

The goal of the experiments was to confirm the possibility of relating skin hydration to the variation of two electrical parameters:

- the relative dielectric constant ($\varepsilon_{r}$), which is derived from the TDR reflectogram and in particular, from the evaluation of the apparent length of the sensing element, $L_{app}$
- by evaluating the electrical conductivity of the skin ($\sigma_{0}$), measured from the steady-state value of the reflection coefficient, evaluated from the TDR reflectogram.

The #1 and #2 sets of experiments were carried out to assess the sensor sensitivity to changes in permittivity and conductivity for different concentrations of these substances in ultrapure water. It should be mentioned that tissue-equivalent liquids are largely employed in specific absorption rate (SAR) compliance assessment tests of wireless devices (i.e., IEC 62209-1). One of the common “recipes” to obtain liquid solutions with dielectric characteristics “mimicking” those of human tissues is to add 50% to 60% of sugar to pure water. If also the conductivity has to be “emulated”, and additional 1% to 6% of salt is further added. The solutions used in the present study cover a variation of dielectric properties, which largely encompasses those of human tissues, and human skin in particular.

After verifying the capability of the system to detect these variations, the set #3 of experiments consisted in analyzing the TDR response in different hydration states of the skin (in particular, of a person’s forearm).

Finally, the experimental session #4 was carried out by placing the SE in contact with a sample of animal hide. Using this sample allowed to bring the hide sample to known values of hydration and experiment on larger hydration ranges.

## 5. Experimental Results

### 5.1. Ultrapure Water and Sugar Solutions

**Figure 3:**
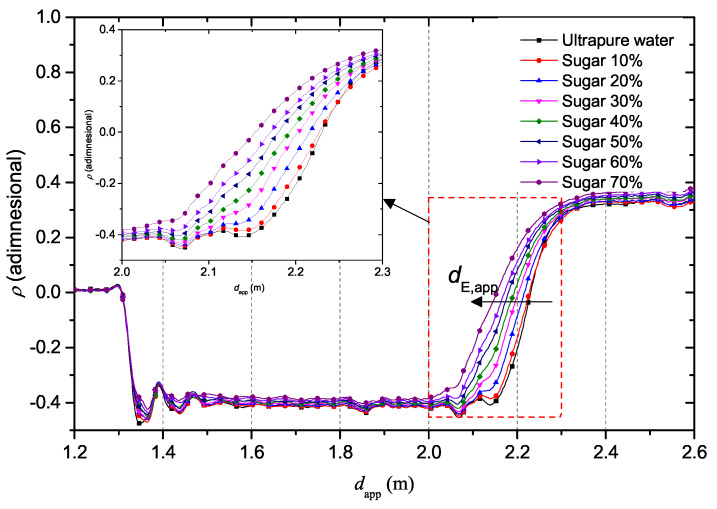
TDR reflectograms for ultrapure water with different mass concentrations of sugar.

The preliminary experiments were conducted with the SE immersed in reference liquid solutions. The first experimental session, TDR responses associated with ultrapure water and ultrapure water with different mass concentrations of sugar were compared. Seven solutions in total were mixed, starting with the lowest value of sugar mass concentration in water (10%), to the highest value (70%), with a 10% step. Figure 3 shows the trend of the TDR reflectograms for the considered solutions.

It can be noticed that the value corresponding to the beginning of the probe, $d_{B,app}$ remains the same because, up to that point, there is no sensing capability. On the other hand, the abscissa value corresponding to the end of the sensing element, $d_{E,app}$, moves towards lower values as the percentage of sugar increases. Overall, the apparent length of the probe decreases with the increase of sugar concentration.

It can be noticed that the apparent distance of the SE tends to decrease as the concentration of sugar increases. Such a decrease reflects a decrease in dielectric permittivity.

This phenomenon depends on the decrease of free water with the increase of sugars dissolved in the solution. In fact, when the sugar percentage is low, then few water molecules bind to the sugar molecules and in this way the free water molecules are still dominant within the solution. As the weight percentage of dissolved sugar increases, however, more and more molecules will be involved in chemical bonds with the sugar and fewer free water molecules will be present in the solution.

The FD results were obtained by converting the TDR data into the FD domain, through a fast Fourier transform (FFT)-based processing. A 15 m-long window guaranteed good accuracy in the estimation of the reflection scattering parameter, $S_{11}\left(f\right)$. This window length allows an optimal trade-off between resolution for the estimation of the apparent length and the possibility of an accurate estimation of the conductivity (which can be retrieved from the portion of reflectogram at “long” distances, practically corresponding to the DC condition).

**Figure 4:**
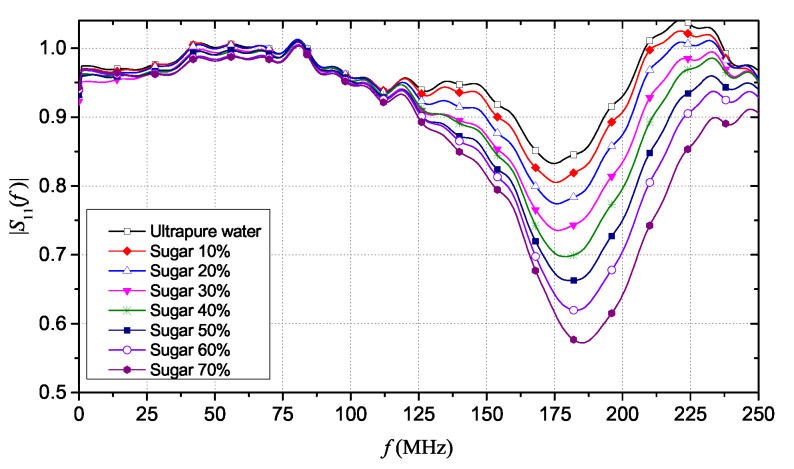
$|S_{11}\left(f\right)|$ results for ultrapure water with different mass concentrations of sugar.

Figure 4 shows the magnitude of $S_{11}\left(f\right)$, in the 0–250 MHz frequency range, which is the range where changes in the $|S_{11}\left(f\right)|$ behavior were more evident. The $|S_{11}\left(f\right)|$ curves exhibit the typical minima: for each solution, the difference in frequency between two consecutive minima (Δf) is directly related to the change in the apparent distance of the SE immersed in the solution [40]. It is possible to note that, as sugar concentration increases, the minima of the curve shift toward higher frequency values and, at the same time, the magnitude of the reflection scattering parameter decreases. With regard to the static conductivity, $\sigma_{0}$, it can be seen that there is no significant variation for the different solutions: in fact, by observing Figure 4, the curves of the $S_{11}\left(f\right)$ are practically superimposed at 0 Hz.

### 5.2. Saline Solution with Sugar

To emulate a material with dielectric parameters similar to those of human skin, a solution with a reference $\sigma_{0}$= 0.10 S/m was prepared and the same different concentrations of sugar were dissolved. Also in this case, mass concentration of sugar ranging from 10% to 70%, with a 10% step were dissolved.

**Figure 5:**
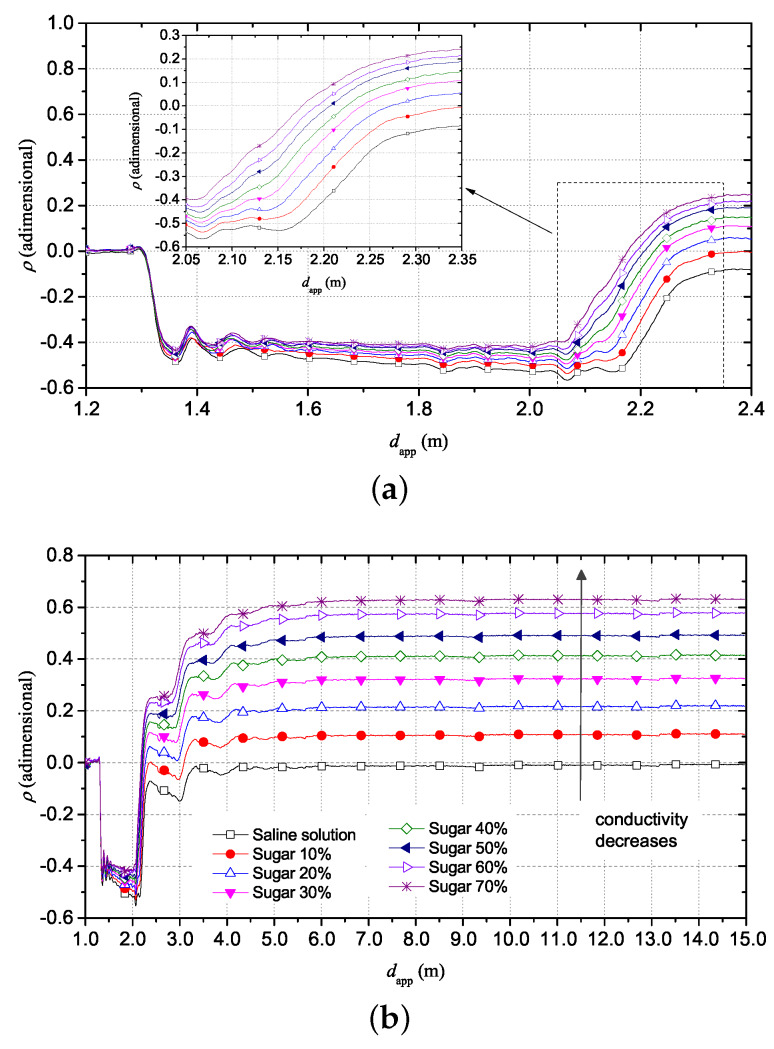
TDR reflectograms for saline solutions ($\sigma_{0}$ = 0.1 S/m) with different mass concentrations of sugar. (**a**) zoom on the reflectograms, with focus on apparent length of SE that decreases with increasing sugar concentration; (**b**) 15 m trend shows the reflection coefficient that increases with decreasing of conductivity.

Figure 5a shows that, as expected, the apparent distance of the SE continues to decrease as the concentration of sugar increases; however, in this case, also the conductivity of the solution decreases. This phenomenon is highlighted by the trend of reflection coefficient ρ that, at long distance, is intrinsically related to the static electrical conductivity of the solution (Figure 5b). As the sugar content increases, the corresponding ρ increases; this happens because the value of $\sigma_{0}$ of the solution decreases.

**Figure 6:**
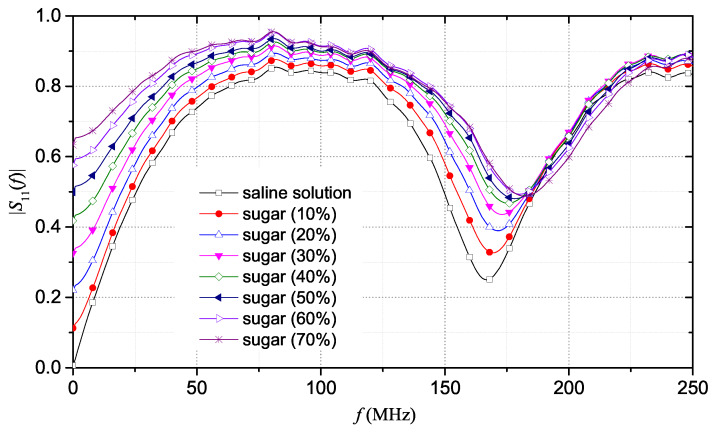
$|S_{11}\left(f\right)|$ of saline solution with different mass concentrations of sugar.

Also in this case, starting from the TDR obtained data, the corresponding $S_{11}\left(f\right)$ information was extrapolated. Figure 6 shows the trend of the $|S_{11}\left(f\right)|$ in the same frequency range analyzed in the previous case. Also in this case, the minima of the curves of $|S_{11}\left(f\right)|$ shift in frequency toward higher frequencies, while the magnitude increases with increasing sugar concentrations in saline solution.

With regard to the static conductivity, in this case it is possible to notice different magnitude values of $|S_{11}\left(f\right)|$ at 0 Hz. In particular, this value increases as the sugar concentration in the saline solution increases and this means that the magnitude of scattering parameter increases as the conductivity of the solutions decreases.

### 5.3. Measurements on Human Forearm Skin

**Figure 7:**
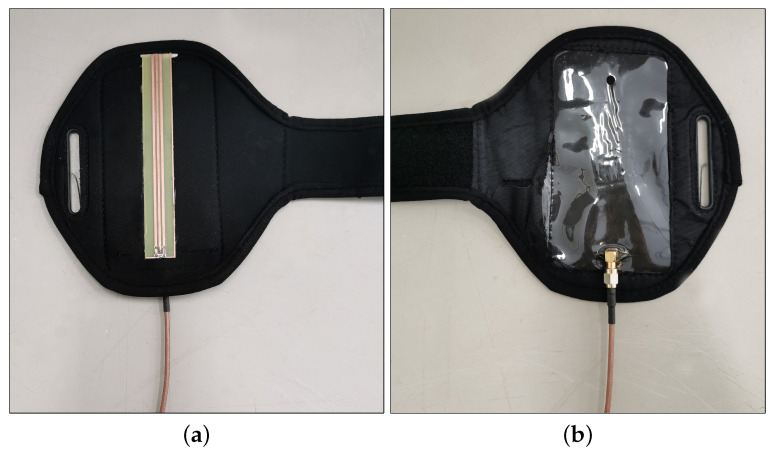
SE attached on the internal surface of a running smartphone armband: (**a**) front; (**b**) back.

**Figure 8:**
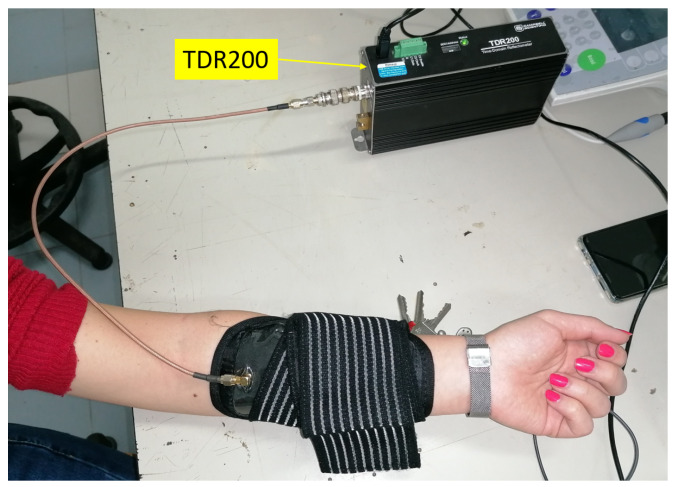
Picture of the experimental setup for measurements on human skin.

After testing the sensitivity of the SE with respect to changes in dielectric permittivity and static conductivity of reference liquid solutions, the SE was attached on the internal surface of a smartphone armband (typically used for running) in order to carry out measurements directly on human skin, as shown in Figure 7. Figure 8 shows a picture of the experimental setup.

Measurements were carried out for four different forearm hydration states. The first condition was that of the forearm in dry condition. Then, a skin lotion was applied to the forearm to increase the skin hydration level and the measurement was repeated. Subsequently, after the lotion had been absorbed almost completely by the skin, the forearm was wetted with water; also in this condition, another TDR measurement was carried out. Finally, the forearm was dried with paper to remove lotion and water residues, and measurement was carried out after few minutes. However, in this situation the forearm was not completely dry.

**Figure 9:**
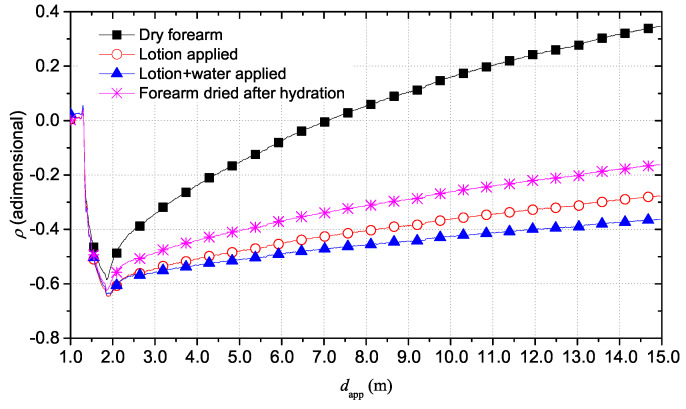
Comparison of TDR reflectograms for the four cases of forearm hydration.

Figure 9 shows the TDR reflectograms associated to the four conditions described above. The results are in good agreement with the described forearm conditions.

**Figure 10:**
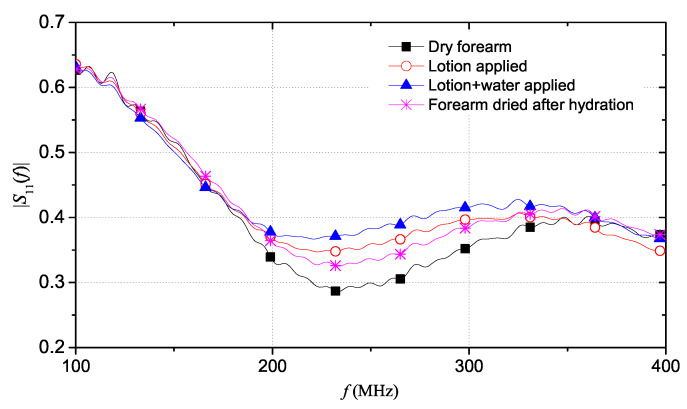
Comparison of the $|S_{11}\left(f\right)|$ for the four cases of forearm hydration.

As aforementioned, the reflection coefficient ρ value at long distances is closely related to the static conductivity $\sigma_{0}$. The dry forearm exhibits the highest value of ρ at 15 m, which corresponds to the lowest value of static conductivity $\sigma_{0}$ compared to the other cases. In fact, the anhydrate skin of forearm has lower content of water and therefore a lower value of conductivity. On the other hand, the highest hydration situation is the case of lotion and water applied on the skin and, predictably, it corresponds to the lowest value of ρ at 15 m, result of the largest increase in conductivity of the skin. In an intermediate situation, there is the case of only lotion applied to the skin, before applying water. In this condition, the forearm is less hydrated than the previous condition and, for this reason, an increase of the ρ value can be observed: this corresponds to a lower conductivity value due to a lower hydration state. Finally, the forearm was dried up and, after several minutes, the measurement was repeated to observe the effects of a moisturizing action over time. Clearly, in this case, the forearm was not completely dry due to strong previous hydration procedure and it was slightly moist. For this, the resulting TDR reflectogram differs from that of the dry forearm. In particular, the reflection coefficient ρ at 15 m is lower than that of the dry forearm but it is higher than the ρ value for the two cases of lotion and water applied to the skin. This means that, in this situation, the effect of hydration still exists but it is lower due to the progressive absorption of moisturizing substances. In fact, the value of ρ corresponds to a lower conductivity with respect to the cases with lotion and cream but a higher conductivity than a completely dry skin. This is all due to the persisting hydrating effect, which tends to disappear over time up to the starting condition. The extrapolation of data in the frequency domain allows to analyze the trend of the $|S_{11}\left(f\right)|$ for the four cases, as shown in Figure 10.

It is interesting to analyze a frequency range of 100–400 MHz. In fact, in this range, it can be seen that $|S_{11}\left(f\right)|$ exhibits distinct changes that follow a specific trend. Additionally, it can be seen that the $|S_{11}\left(f\right)|$ value progressively increases when skin hydration increases, due to the increase of the electrical conductivity of the skin when it is progressively hydrated. In addition, as evidenced by previous tests, a decrease in water in the skin, due to a decrease in hydration, involves a shift of $|S_{11}\left(f\right)|$ minima towards higher frequencies. This phenomenon is due to changes in the dielectric constant of the skin at different hydration states.

Finally, it is worth noting that a single TDR measurement takes approximately a couple of minutes. Therefore, in view of practical monitoring applications, this can be considered as the time resolution that can be achieved between two consecutive TDR measurements on a patient. This should suffice to ensure adequate online monitoring.

### 5.4. Measurements on Animal Hide

To test the sensitivity of the proposed monitoring system to wider ranges of skin hydration, the final experiment was conducted on a piece of animal hide. This was done in order to emulate human tissue. Moreover, as aforementioned, using a hide sample allowed to bring the sample to known values of hydration and experiment and hydration larger ranges.

In addition, this experiment allowed to assess the relationship between the electrical quantity and hydration, we have carried out measurements on a piece of animal hide brought at known reference values of hydration. As a result, it was possible to retrieve a calibration curve relating $\varepsilon_{r}$ to the hydration of the hide. The hide sample (dry weight of 8.4 g), was immersed in water until it reached progressively increasing water content values. The water-saturation condition corresponds to 70% of water in the sample while the other measurements correspond to 64% and 58% of water in the sample, respectively. The moisture content values reached by the hide sample were evaluated through the gravimetric method.

**Figure 11:**
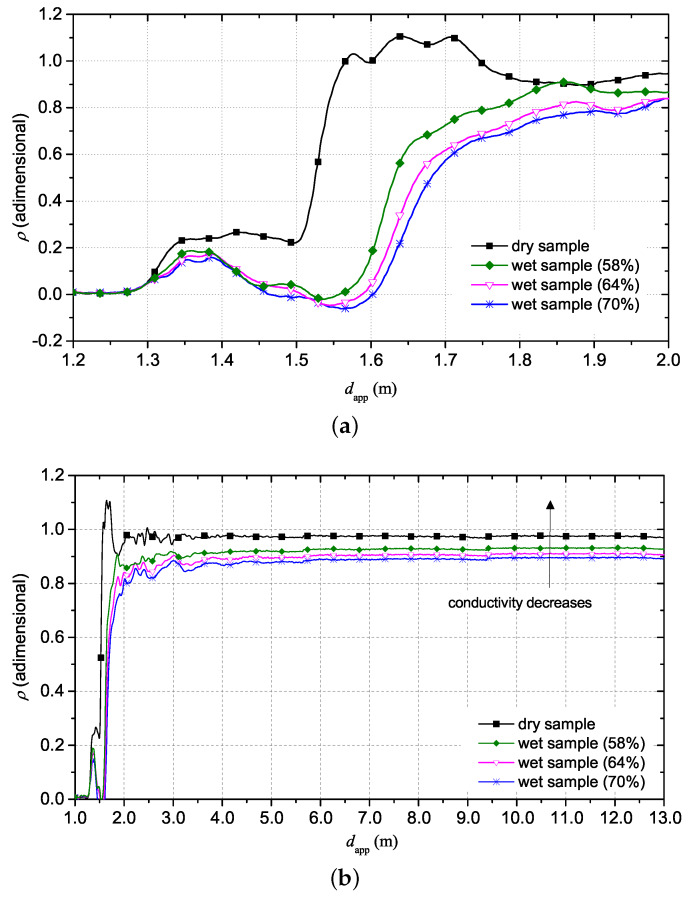
TDR reflectograms for animal hide sample. (**a**) reflectograms at short distance; (**b**) reflection coefficient at long distance with focus on the trend of electrical conductivity.

Figure 11 shows that the reflection coefficient ρ, at long distances, increases with decreasing of water content in the sample and, consequently, with decreasing of static conductivity $\sigma_{0}$. In addition, once piece of hide dries, it gradually loses its water content and, for this reason, the permittivity decreases. This aspect can be seen in the decreasing of apparent length of SE as sample dries.

## 6. Conclusions

In this study, the feasibility of a TDR-based wearable skin hydration sensing system for real-time, continuous monitoring of skin hydration level was investigated. The feasibility of the proposed system was assessed through experimental tests on reference liquid solutions, directly on human skin and on tanned animal hide. The proposed system guarantees good accuracy, low cost, possibility of remote control and portability that make it particularly attractive for practical medical applications, in particular for sick people or older people with walking difficulties. The proposed hydration monitoring system can represent an important resource to help patients in monitoring their health status. The implementation of such a system is quite promising, as it would allow a substantial improvement of the remote control of diseases.

This system can be further miniaturized and made completely portable, in order to achieve a new wearable skin hydration monitoring system based on TDR technique. To ensure wearability of the system, the authors are working on a miniaturized version of a TDR measuring instrument that could be attached directly to the armband. The miniaturized TDR instrument will be based on FPGA technology, similarly to the one proposed in [43]. In turn, the TDR measuring instrument will transmit data wirelessly, for example, to a remote monitoring station. The goal is to guarantee complete freedom of movement to the user.

The upcoming work will regard the identification of the relationship between physiological parameters and the TDR-measured quantities (for example, the frequency value of the peak of the $|S_{11}\left(f\right)|$; the value of $|S_{11}\left(f\right)|$ at 0 Hz; or the electrical conductivity calculated from the long-distance value of ρ in the reflectogram). To this purpose, the next step will require to verify the proposed system on a number of voluntary subjects whose physiological parameters will be determined also through comparative, reference measurement system. This will allow to validate the proposed system also from a metrological point of view.
